# Prognostic Value of Metabolic Syndrome in Patients With Non-ST Elevated Myocardial Infarction Undergoing Percutaneous Coronary Intervention

**DOI:** 10.3389/fcvm.2022.912999

**Published:** 2022-06-23

**Authors:** Li-Hong Zhao, Yin Liu, Jian-Yong Xiao, Ji-Xiang Wang, Xiao-Wei Li, Zhuang Cui, Jing Gao

**Affiliations:** ^1^Graduate School, Tianjin Medical University, Tianjin, China; ^2^Cardiac Function Department, The First Affiliated Hospital of Baotou Medical College, Inner Mongolia University of Science and Technology, Baotou, China; ^3^Department of Cardiology, Tianjin Chest Hospital, Tianjin, China; ^4^School of Public Health, Tianjin Medical University, Tianjin, China; ^5^Tianjin Cardiovascular Institute, Tianjin Chest Hospital, Tianjin, China; ^6^Thoracic Clinical College, Tianjin Medical University, Tianjin, China

**Keywords:** metabolic syndrome, non-ST elevated myocardial infarction, prognostic value, percutaneous coronary intervention, cohort study

## Abstract

**Objective:**

We aim to investigate the prognostic effects of metabolic syndrome (MS) on patients with non-ST elevated myocardial infarction (NSTEMI) after percutaneous coronary intervention (PCI).

**Methods:**

Patients with NSTEMI undergoing PCI were consecutively collected. According to the presence or absence of MS, they were divided into two groups and followed up for 1 year. The endpoint was major adverse cardiovascular events (MACE), including all-cause death, unstable angina hospitalization, heart failure (HF) hospitalization, non-fatal recurrent myocardial infarction (MI), and target lesion revascularization. Also, six subgroups were made according to gender, age, left ventricular ejection fraction (LVEF), Global Registry of Acute Coronary Events (GRACE) score, hypersensitive troponin (hsTNT), and several diseased vessels. Cox proportional hazard model was adopted to analyze the effect of MS on MACE in all the patients and different subgroups.

**Results:**

A total of 1,295 patients were included in the current analysis and 660 (50.97%) of them had MS. About 88 patients were lost to follow-up, and the overall average follow-up was 315 days. MS was an independent risk factor for MACE (HR 1.714, CI 1.265–2.322, *p* = 0.001), all-cause death, heart failure (HF) hospitalization, and non-fatal recurrent MI. In the MS component, BMI ≥28 kg/m^2^ was positively associated with MACE. Subgroup analysis indicated the prognostic value of MS was more striking for patients with the following: age of >60, LVEF of ≤40%, GRACE of >140, multivessel disease, or hsTNT of >0.1 ng/ml.

**Conclusions:**

The MS was a robust adverse prognostic factor in patients diagnosed with NSTEMI, especially among those of older age and at higher ischemic risk. A BMI of ≥28 kg/m^2^ independently predicted the occurrence of MACE. Prognosis may be improved by controlling abdominal obesity.

## Introduction

Metabolic syndrome (MS) is a disease state that involves multiple metabolic abnormalities and is closely related to cardiovascular disease (CVD), mainly including central obesity, hypertension, dyslipidemia, and diabetes or abnormal glucose tolerance. Furthermore, MS is associated with future incidence of diabetes and CVD and subsequent adverse cardiovascular events (1, 2). The increasing incidence of the syndrome worldwide, as well as the number of people with MS, will continue to rise, which has made it a serious public health problem.

As a serious cardiac emergency, patients with non-ST elevated myocardial infarction (NSTEMI) are likely to have more comorbidities and a worse prognosis than patients with ST-elevation myocardial infarction (STEMI) (3). Hospitalization rates for NSTEMI are increasing, and a transition from STEMI to NSTEMI in acute myocardial infarction (AMI) has been observed in China (4, 5). However, studies on the effects of MS on CVD were mostly focused on acute coronary syndrome (ACS) and acute STEMI and those results were controversial. Studies in non-ST elevation acute coronary syndrome (NSTEACS) or patients with NSTEMI are still scarce. Beyond that, controversy exists about whether there is a correlation between each component and prognosis, and which component is more important. As for patients with NSTEMI undergoing PCI, it is unclear whether the presence or absence of MS affects the occurrence of major adverse cardiovascular events in patients with different clinical and angiographic outcomes. Therefore, the current study aimed to evaluate the impact of MS and its components on cardiovascular outcomes in patients with NSTEMI undergoing PCI within 12 months after discharge. In addition, we further explored the prognostic value of MS in different subgroups of patients with NSTEMI.

## Methods

### Study Design and Participants

From January 2018 to December 2019, consecutive patients with NSTEMI undergoing percutaneous coronary intervention (PCI) in Tianjin Chest Hospital were included. The inclusion criteria are as follows: (1) Age ≥18 years old; (2) In line with the diagnostic criteria of NSTEMI, cardiac troponin and/or CKMB above the 99th percentile of healthy individuals, and the following with at least one: (i) ST-segment depression or T wave inversion on ECG; (ii) Chest pain persists longer than 30 min; (3) Onset time <30 days; and (4) Patients undergoing coronary angiography and treated with PCI during the period of admission. The exclusion criteria are as follows: (1) Myocardial infarction (MI) patients if those ECG were persistent ST-segment elevation during diagnosis and treatment; (2) Chest pain caused by non-cardiac diseases, such as aortic dissection and pulmonary embolism, etc.; (3) Active bleeding, severe thrombocytopenia, severe liver or kidney diseases, malignant tumors, etc.; and (4) Lack of diagnostic data related to MS. After screening and meeting the above inclusion and exclusion criteria, 1,295 patients were eventually included. The study was performed by the Declaration of Helsinki and was approved by the Ethics Committee of Tianjin Chest Hospital (No. 2018KY-010-01). All included patients provided signed informed consent forms before study participation.

### Definition of Metabolic Syndrome

The MS was defined according to a scientific statement (6) from the American Heart Association/National Heart, Lung, and Blood Institute (AHA/NHLBI). Patients with 3 or more of the following risk factors were considered to have MS: (i) Central obesity: waist circumference (WC) of ≥102 cm in men and ≥88 cm in women. (ii) hypertriglyceridemia (triglycerides ≥1.7 mmol/L or on drug treatment for elevated triglycerides), (iii) low HDL-C (<1.03 mmol/L in men and <1.29 mmol/L in women or on drug treatment for reduced HDL-C), (iv) arterial hypertension [≥130 mmHg systolic blood pressure (SBP) or ≥85 mmHg diastolic blood pressure (DBP) or antihypertensive therapy], and hyperglycemia (fasting blood glucose ≥5.6 mmol/L or antidiabetic treatment). Since the patient's WC was not available, we used BMI as a surrogate parameter for WC, which had been adopted and verified in previous studies (7, 8). We utilized the BMI of ≥28 kg/m^2^ as a diagnostic criterion of obesity proposed by the Working Group on Obesity of China (WGOC) (9).

### Clinical and Biochemical Measurements

The basic data of gender, age, BMI, and previous medical history of all patients were recorded on admission, and the admission conditions of the patients were evaluated, including sitting blood pressure (measured by senior doctors on the non-dominant arm supported by the heart level), Killip class, and Global Registry of Acute Coronary Events (GRACE) score. The emergency laboratory indexes of admission, such as creatine kinase (CK), creatine kinase MB (CK-MB), hypersensitive troponin T (hsTnT), etc., were recorded. After fasting overnight for 12 h, fasting blood glucose (FBG), triglyceride (TG), high-density lipoprotein cholesterol (HDL-C), and low-density lipoprotein cholesterol (LDL-C) total cholesterol were measured. GRACE was calculated for all patients on admission using eight variables, including age, systolic blood pressure, heart rate, serum creatinine, Killip class, cardiac arrest, elevated cardiac biomarkers (hsTnT), and ST deviation. The calculator used is available at (http://www.outcomes-umassmed.org/GRACE/). Ejection fraction (EF) was assessed by echocardiography in the first week after AMI according to the clinical standards and current echocardiography guidelines (10). Coronary angiography and PCI were performed by two cardiologists with qualifications for coronary artery diagnosis and treatment simultaneously according to the international standards and guidelines (11). Postoperative antiplatelet therapy with 100 mg/d of aspirin and 75 mg/d of clopidogrel, or 90 mg of ticagrelor twice per day is recommended for at least 1 year.

### Study Endpoint and Follow-Up

All patients were followed up for 1 year after discharge, including outpatient visits, telephone interviews, and the recording of recurrent all-cause events by trained nurses or cardiologists, after the initial appointment.

The endpoint was MACE, including all-cause death, unstable angina (UA) hospitalization, heart failure (HF) hospitalization, non-fatal recurrent MI, and target lesion revascularization (TLR). TLR therapy was for either lesion with ischemia symptoms or event-driven, including PCI and coronary artery bypass grafting (CABG). In the case of more than one clinical event in the same patient, only the first event was considered, and follow-up was discontinued. Patients lost to follow-up or those without MACE were treated as censored.

### Statistical Analysis

Continuous variables were expressed as mean ± standard deviation (SD) or median with an interquartile range based on its distribution, and categorical variables were expressed as frequency and percentage. For comparison of continuous variables, the independent Student's *t*-test or Mann-Whitney *U*-test was used, and for comparison of categorical variables, the chi-square test or Fisher's exact tests was used, where appropriate. Event-free survival curves were analyzed by the Kaplan-Meier method, and a comparison between curves was carried out by the log-rank test. Multivariate Cox regression analysis was performed to evaluate the impact of MS and its five components on MACE in all enrolled patients with NSTEMI and various subgroups. The covariates included in the multivariable model were as follows: age, gender, history of smoking, family history of CVD, Killip ≥II, LVEF of ≤40%, multi-vessel disease, ACEI/ARB, and Beta-blocker. All statistical tests were 2-sided with *p* < 0.05 considered statistically significant. SPSS software (version 25.0, SPSS, Inc., Chicago, Illinois, USA) was used for statistical analysis. Subgroups were classified: 1,295 patients with NSTEMI by male vs. female; age of ≤60 vs. age of >60; LVEF of ≤40% vs. LVEF of >40%; low/medium (≤140) vs. high (>140) GRACE score; single-vessel disease (SVD) vs. multivessel disease (MVD); and hsTNT of ≤0.1 ng/ml vs. hsTNT of >0.1 ng/ml, respectively.

## Results

### Clinical Characteristics

Among 1,295 patients with NSTEMI, there were 660 patients with MS (50.97%) and 635 patients without MS (49.03%). About 88 patients were lost to follow-up, and the overall average follow-up was 315 days. Women accounted for 36.7% in the MS group and 23.6% in the non-MS group, with statistical significance between the two groups. Compared with the non-MS group, the MS group had a higher body mass index (BMI), an average lower LVEF, and a higher hypersensitive C-reactive protein (hs-CRP) level. Among the components of MS, obesity, diabetes, and hypertension were more prevalent, HDL-C was lower, and TG and FBG were higher in patients with MS, as compared to those without MS, as expected. There was no significant difference between the two groups in age, family history of CVD, history of smoking, previous PCI, previous CABG, and the use of medications. There was no difference in GRACE score between the two groups. No significant differences were observed between the groups concerning the proportion of SVD and MVD and the application of intra-aortic balloon pump or ventilator (Table 1).

**Table 1:** Baseline patient characteristics according to metabolic syndrome.

**Variables**	**MS (-) (*n* = 635)**	**MS (+) (*n* = 660)**	***P***
Age (years)	65 (57.72)	66 (57.73)	0.552
Female, gender *n* (%)	150 (23.6)	242 (36.7)	<0.001
BMI (%) <24 (kg/m^2^), *n* (%)	401 (63.1)	241 (36.5)	<0.001
24–28 (kg/m^2^), *n* (%)	165 (26.0)	185 (28.0)	
≥28 (kg/m^2^), *n* (%)	69 (10.9)	234 (35.5)	
BMI (kg/m^2^)	22.9 (21.5, 24.6)	27.2 (22.3, 29.0)	<0.001
**Medical history**			
Hypertension, *n* (%)	318 (50.1)	557 (84.4)	<0.001
Diabetes, *n* (%)	110 (17.3)	316 (47.9)	<0.001
Previous MI, *n* (%)	105 (16.5)	132 (20.0)	0.107
Previous stroke, *n* (%)	158 (24.9)	176 (26.7)	0.463
Previous PCI, *n* (%)	94 (14.8)	114 (17.3)	0.226
Previous CABG, *n* (%)	27 (4.3)	40 (6.1)	0.142
Family history of CVD, *n* (%)	62 (9.8)	72 (10.9)	0.499
Smoking, *n* (%)	384 (60.5)	430 (65.2)	0.081
**Admission**			
Systolic blood pressure (mmHg)	130 (120, 145)	135 (122, 150)	<0.001
Diastolic blood pressure (mmHg)	75 (69.84)	76 (70.85)	0.142
Killip class, *n* (%)			0.481
I	542 (85.4)	564 (85.5)	
II	79 (12.4)	73 (11.1)	
III	11 (1.7)	19 (2.9)	
IV	3 (0.5)	4 (0.6)	
Killip ≥II, *n* (%)	93 (14.6)	96 (14.5)	0.959
**Laboratory**			
LVEF ≤40%, *n* (%)	84 (14.3)	85 (13.9)	0.825
LVEF (%)	55 (47, 59)	54 (45, 58)	0.008
Fasting plasma glucose (mmol/L)	5.21 (4.72, 5.95)	6.63 (5.64, 8.76)	<0.001
Triglyceride (mmol/L)	1.30 (1.04, 1.58)	1.96 (1.50, 2.52)	<0.001
Total cholesterol (mmol/L)	4.36 (3.73, 5.09)	4.50 (3.79, 5.14)	0.1
HDL-C (mmol/L)	1.09 (0.93, 1.30)	0.90 (0.78, 1.01)	<0.001
LDL-C (mmol/L)	2.93 (2.28, 3.55)	2.99 (2.37, 3.60)	0.275
hsCRP (mg/L)	4.71 (1.72, 16.36)	5.97 (2.65, 15.96)	0.002
NT-proBNP (pg/ml)	896.9 (380, 2287)	907 (349.6, 2961.75)	0.627
hsTnT (ng/mL)	0.57 (0.24, 1.24)	0.54 (0.24, 1.23)	0.741
CK (U/L)	222.5 (110, 519.5)	208.5 (103.5, 455)	0.299
CK-MB (U/L)	28 (17, 55)	26 (16, 47)	0.255
**CAG and treatment**			
Single-vessel disease, *n* (%)	147 (23.1)	131 (19.8)	0.148
Double-vessel disease, *n* (%)	161 (25.4)	162 (24.5)	0.737
Triple-vessel disease, *n* (%)	312 (49.1)	352 (53.3)	0.131
Left main, *n* (%)	94 (14.8)	95 (14.4)	0.835
Multi-vessel disease, *n* (%)	488 (76.9)	529 (80.2)	0.148
Grace Grade, *n* (%)			0.454
<109, *n* (%)	192 (30.2)	206 (31.2)	
109–140, *n* (%)	246 (38.7)	234 (35.5)	
>140, *n* (%)	197 (31.0)	220 (33.3)	
IABP, *n* (%)	19 (3.0)	29 (4.4)	0.182
Respirator, *n* (%)	72 (11.3)	81 (12.3)	0.603
**Baseline medication**			
DAPT, *n* (%)	625 (98.4)	649 (98.3)	0.896
Beta-blocker, *n* (%)	478 (75.3)	522 (79.1)	0.102
ACEI/ARB, *n* (%)	394 (62.0)	429 (65.0)	0.270
Statin, *n* (%)	612 (96.4)	638 (96.7)	0.777
Anticoagulants, *n* (%)	624 (98.3)	649 (98.3)	0.927

*ACEI, angiotensin-converting enzyme inhibitor; ARB, angiotensin receptor blocker; BMI, body mass index; CABG, coronary artery bypass graft; CK, creatine kinase; CK-MB, creatine kinase MB; CVD, cardiovascular disease; DAPT, dual antiplatelet therapy; GRACE, Global Registry of Acute Coronary Events; HDL-C, high-density lipoprotein cholesterol; hsCRP, high sensitivity C-reactive protein; hsTnT, hypersensitive troponin T; IABP, intra-aortic balloon pump; LDL-C, low-density lipoprotein cholesterol; LVEF, left ventricular ejection fraction; MI, myocardial infarction; MS, metabolic syndrome; NT-proBNP, N-Terminal pro-brain natriuretic peptide; PCI, percutaneous coronary intervention*.

### Clinical Prognosis

**Figure 1:**
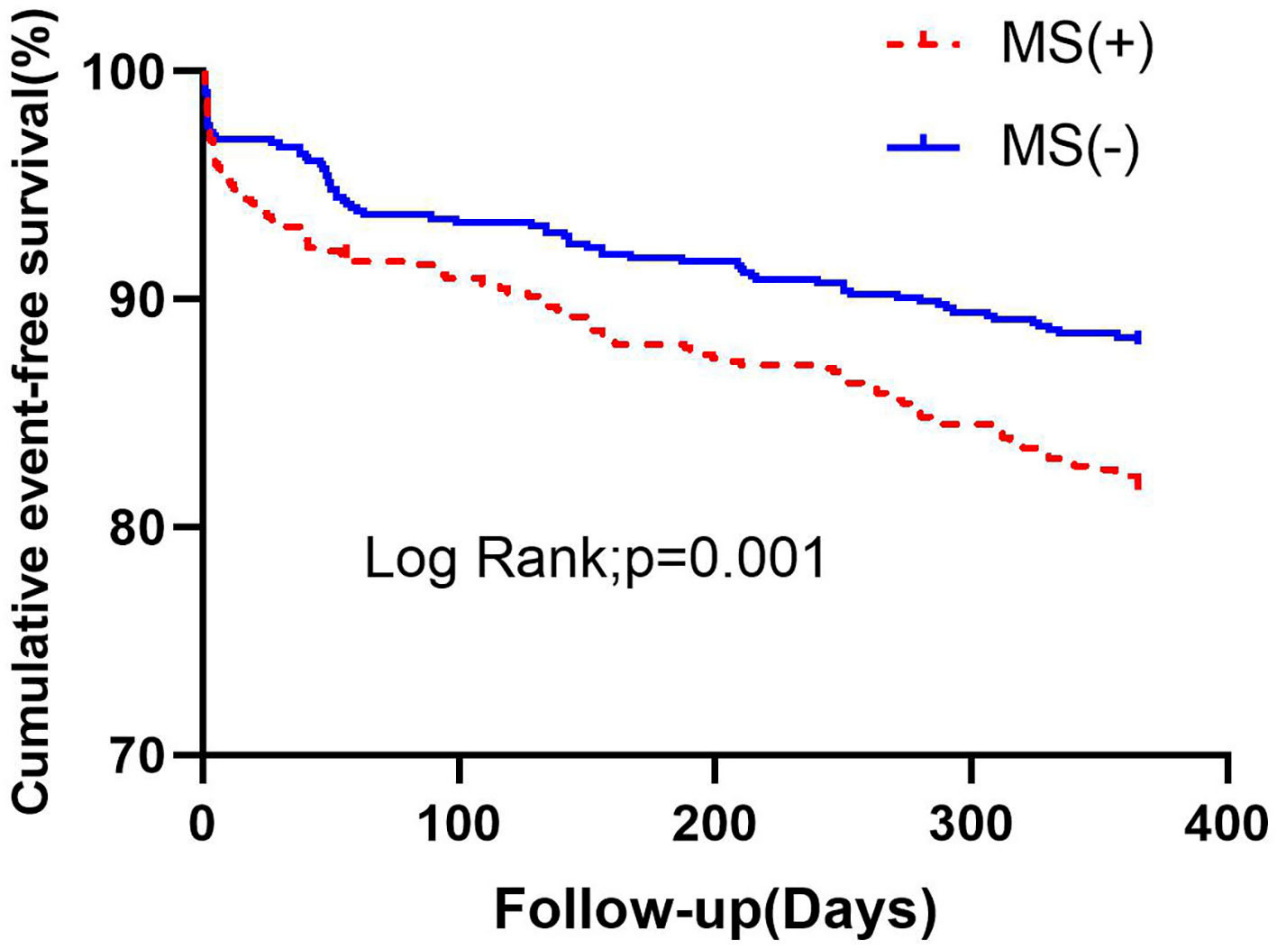
Kaplan-Meier survival analysis of cumulative event-free survival curves within 1 year according to MS. MS, metabolic syndrome.

Procedural success was achieved in all patients. Throughout the follow-up period, patients with MS had significantly higher rates of MACE than those without MS. Of the 1,295 people, 197 (15.2%) were involved in the MACE. There were 52 (4%) patients with all-cause death, 56 (4.3%) patients with UA hospitalization, 76 (5.9%) patients with HF hospitalization, 26 (2%) patients with non-fatal recurrent myocardial infarction (MI), and 5 (.4%) patients with TLR. During 1-year follow-up, it was found that there was a significant difference in MACE between the two groups (18.3 vs. 12%, *p* = 0.001), mainly due to the incidence of all-cause death, HF hospitalization, and non-fatal recurrent MI, which were significantly higher in the MS group than that of the non-MS group (5.3 vs. 2.7%, *p* = 0.016; 7.3 vs. 4.4%, *p* = 0.028; 3.2–0.8%, *p* = 0.002, Table 2). There was a significant difference in the Kaplan-Meier curve between the two groups (*p* = 0.001) (Figure 1).

**Table 2:** Comparison of clinical outcomes between two groups.

**Variables**	**MS (-) (*n* = 635)**	**MS (+) (*n* = 660)**	***P***
MACE	76 (12)	121 (18.3)	0.001
All-cause death	17 (2.7)	35 (5.3)	0.016
UA hospitalization	29 (4.6)	27 (4.1)	0.674
HF hospitalization	28 (4.4)	48 (7.3)	0.028
Non-fatal recurrent MI	5 (0.8)	21 (3.2)	0.002
TLR	1 (0.2)	4 (0.6)	0.193

*HF, heart failure; MACE, major adverse cardiovascular events; MI, myocardial infarction; MS, metabolic syndrome; TLR, target lesion revascularization; UA, unstable angina*.

### Association MS and Its Components With MACE

The COX regression analysis showed that MS was an independent risk factor for MACE [HR 1.714, 95% confidence interval (CI) 1.265–2.322, *p* = 0.001], mainly due to the increased risk of all-cause death, HF hospitalization, and non-fatal recurrent MI (HR 2.184, 95% CI 1.137–4.197, *p* = 0.019; HR 1.662, 95% CI 1.031–2.680, *p* = 0.037; HR 3.621, 95% CI 1.324–9.904, *p* = 0.012) (Table 3).

**Table 3:** Multivariate COX regression analysis of each adverse event.

**Variables**	**HR (95% CI)**	***P***
MACE	1.714 (1.265–2.322)	0.001
All-cause death	2.184 (1.137–4.197)	0.019
UA hospitalization	0.890 (0.508–1.559)	0.683
HF hospitalization	1.662 (1.031–2.680)	0.037
Non-fatal recurrent MI	3.621 (1.324–9.904)	0.012
TLR	5.215 (0.545–49.861)	0.152

*CI, confidence interval; HF, heart failure; HR, hazard ratio; MACE, major adverse cardiovascular events; MI, myocardial infarction; MS, metabolic syndrome; NSTEMI, non-ST elevated myocardial infarction; UA, unstable angina; TLR, target lesion revascularization*.

**Figure 2:**
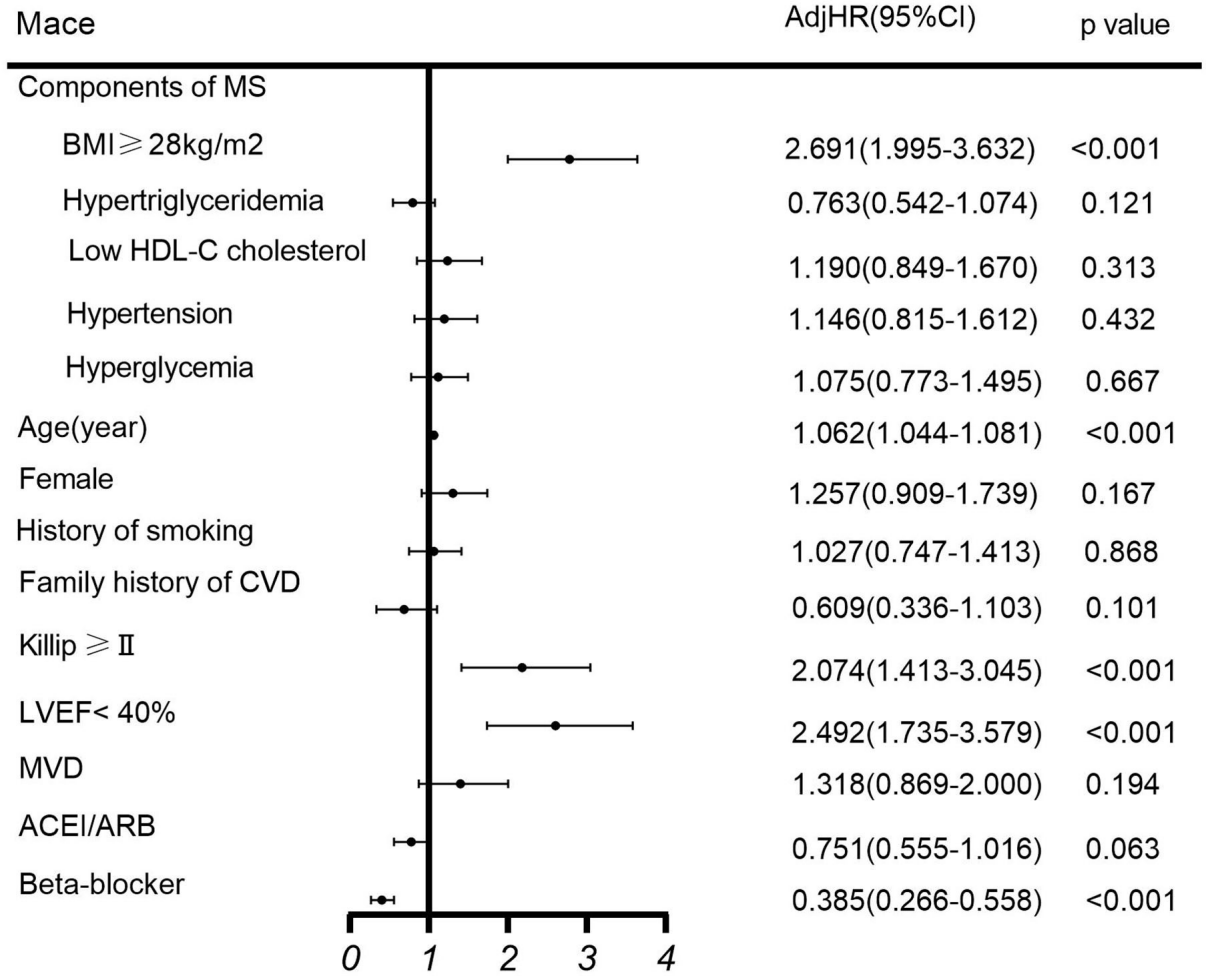
Multivariate COX regression analysis of MACE. ACEI, angiotensin-converting enzyme inhibitor; ARB, angiotensin receptor blocker; BMI, body mass index; CI, confidence interval; CVD, cardiovascular disease; HDL-C, high-density lipoprotein cholesterol; HR, hazard ratio; LVEF, left ventricular ejection fraction; MACE, major adverse cardiovascular events; MS, metabolic syndrome; MVD, multivessel disease.

Among the MS components, multivariate COX regression analysis showed that BMI of ≥28 mg/m^2^ was an independent risk factor for MACE (HR 2.691, 95% CI 1.995–3.632, *p* < 0.001) in patients with NSTEMI undergoing PCI, while other components of MS are not (Figure 2).

### Association of MS With MACE in Subgroups

**Figure 3:**
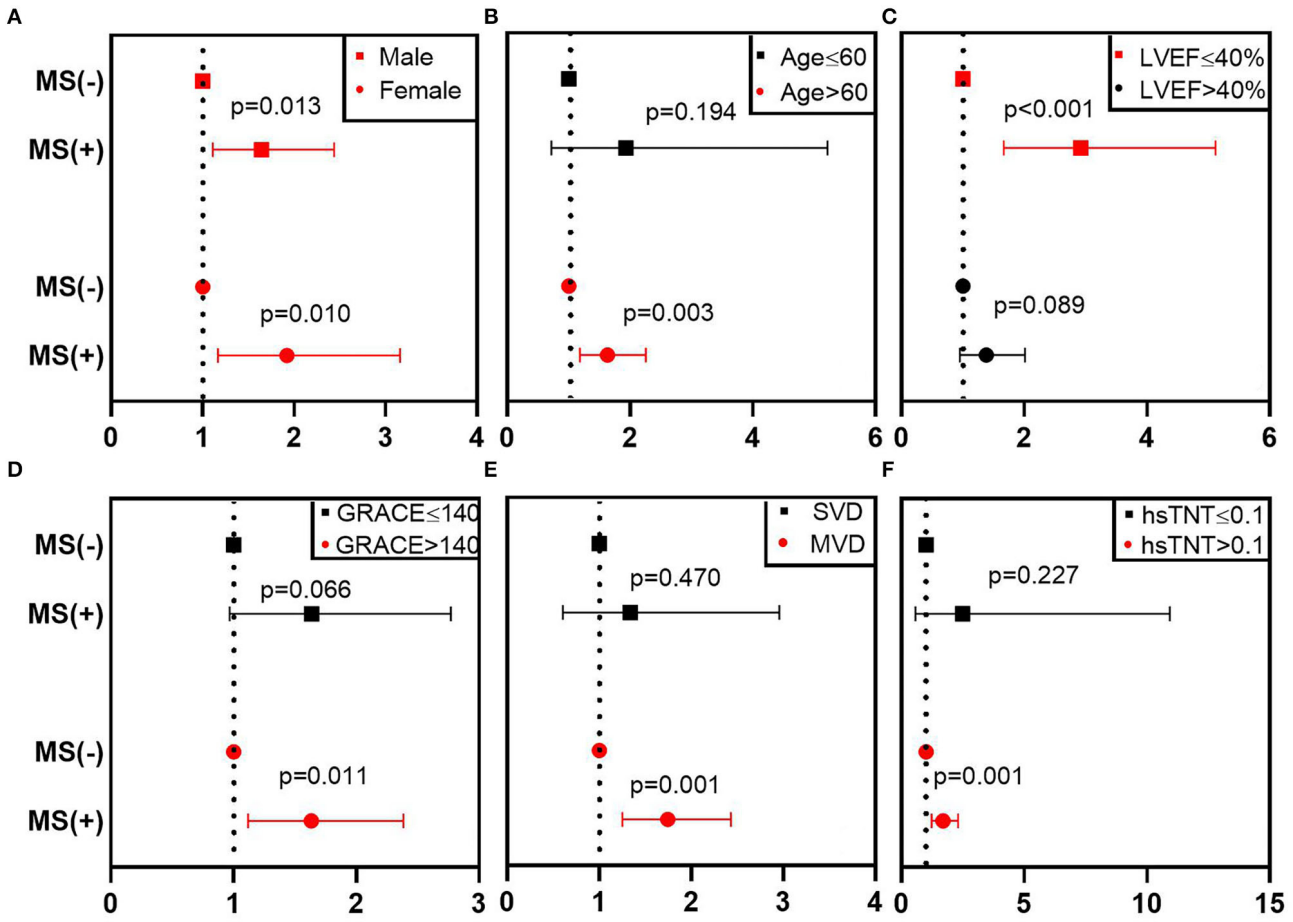
Subgroup analysis of MS on MACE in different patients. This figure shows the adjusted HR (95% CI) of the subgroup analysis from the multivariable Cox model. **(A–F)** Represent the subgroups according to different gender, age, LVEF, GRACE, number of stenosis vessels, hsTNT. The red line denotes a statistically significant subgroup. CI, confidence interval; GRACE, Global Registry of Acute Coronary Events; hsTnT, hypersensitive troponin T; HR, hazard ratio; LVEF, left ventricular ejection fraction; MACE, major adverse cardiovascular events; MS, metabolic syndrome; MVD, multivessel disease; SVD, single-vessel disease.

Furthermore, subgroup analysis of MS on MACE was performed using a Cox model. We found that MS was a robust factor across different genders, and the prognostic value was more evident for patients who have the following: age of >60 (HR 1.633, 95% CI 1.182–2.256, *p* = 0.003), LVEF of ≤40% (HR 2.922, 95% CI 1.668–5.119, *p* < 0.001), GRACE of >140 (HR1.633, 95% CI 1.118–2.385, *p* = 0.011), MVD (HR 1.744, 95% CI 1.251–2.432, *p* = 0.001), or hsTNT of >0.1 ng/ml (HR 1.689, 95% CI 1.237–2.307, *p* = 0.001) (Figure 3; Table 4).

**Table 4:** Multivariate COX regression analysis of each subgroup.

**Variables**		**HR (95% CI)**	***P***
Gender	Male	1.644 (1.110–2.437)	0.013
	Female	1.921 (1.168–3.157)	0.010
Age	≤60	1.931 (0.715–5.218)	0.194
	>60	1.633 (1.182–2.256)	0.003
LVEF	<40%	2.922 (1.668–5.119)	<0.001
	≥40%	1.384 (0.952–2.013)	0.089
GRACE	≤140	1.637 (0.968–2.770)	0.066
	>140	1.633 (1.118–2.385)	0.011
Number of stenosis vessels	SVD	1.339 (0.606–2.957)	0.470
	MVD	1.744 (1.251–2.432)	0.001
hsTNT	≤0.1	2.488 (0.567–10.925)	0.227
	>0.1	1.689 (1.237–2.307)	0.001

*CI, confidence interval; GRACE, Global Registry of Acute Coronary Events; HR, hazard ratio; hsTnT, hypersensitive troponin T; LVEF, left ventricular ejection fraction; MACE, major adverse cardiovascular events; MS, metabolic syndrome; SVD, single-vessel disease; MVD, multivessel disease*.

## Discussion

To the best of our knowledge, this is the first report to show the prognostic impact of MS in patients with NSTEMI undergoing PCI. The main findings were: (1) The prevalence rate of MS in patients with NSTEMI was 50.97%. Compared with the non-MS group, the MS group had more females; (2) After a year of follow-up, MACE in the MS group was significantly higher than that in the non-MS group, and MS was an independent risk factor for MACE, mainly due to the increased risk of all-cause death, HF hospitalization, and non-fatal recurrent MI; (3) BMI of ≥28 kg/m^2^ in the MS components independently predict the occurrence of MACE; and (4) Subgroup analysis suggested that MS was a robust factor across different genders. However, the prognostic value was more evident for patients with the following: older than 60 years; LVEF ≤40%, GRACE >140, and MVD or hsTNT >0.1 ng/ml.

It is not surprising to find that the prevalence of MS in patients with NSTEMI was higher than that from population-based surveys. A survey indicated that 33.9% (31% in males and 36.8% in females) had MS in mainland China in 2010 (12). In patients with established CVD, the prevalence of MS ranged from 29 to 66% in previous reports (13–17). A single-center, prospective, and observational study was conducted by Sinha et al. showed that patients with NSTEACS were more common than STEMI in MS group (18). Al-Rasadi et al. also found that MS was more likely associated with NSTEMI (16). The prevalence of MS as seen in our study was similar when compared with previous reports. In our study, there were more women in the MS group than in the non-MS group. This finding was consistent with previous research (19).

After 1 year follow-up, we found that MS was independently associated with MACE, and some previous studies had similar results (17, 20, 21). We found that this was due to the increased risk of all-cause death, HF hospitalization, and non-fatal recurrent MI, which has been similarly reported in previous studies (22–25).

In our study of MS components, obesity was the only independent risk factor for MACE, while other components including hyperglycemia were not. Patients with obesity are in a state of chronic inflammation and pre-thrombotic state, which plays an important role in the process of atherosclerosis and will lead to adverse cardiovascular events (26). It has been confirmed by previous research that abdominal obesity is independently associated with CVD (27, 28). Visceral fat was almost a good predictor of MS (29). It is now generally accepted that the increase in obesity is the most important in the five components of the MS, because it provides the core of the other four components (30). The obesity paradox (31) had not been observed in our study. Previous research has suggested that this obesity paradox may exist due to BMI being not a good indicator of obesity in general obesity because it does not distinguish between fat and lean mass, and may rely more on measurements of fat distribution than the amount of body fat (28). Incidence and outcomes of CVD vary by nations and country of origin (32). The prevalence of CVD is particularly high among South Asians [people originate from the Indian subcontinent (SA)], including SA immigrants living outside the Asian subcontinent, also known as the SA diaspora (33). SA had a larger percentage of body fat and larger visceral adipose tissue at a given BMI compared with other ethnic groups. Asians, including Chinese, have more visceral fat and less skeletal muscle mass than westerners (30). Therefore, the association between BMI and CVD risk may be underestimated in Asians (34). Therefore, the obesity paradox is not evident in the Chinese population. A similar study extends the observation made by Chen et al., which demonstrated (35) that obesity (BMI ≥28 kg/m^2^) increased the risk of all-cause mortality in Chinese patients with CVD. Our study found that hyperglycemia in MS component was not an independent risk factor for MACE. This finding was in accordance with Grundy SM's conclusion that the independent contribution of hyperglycemic status to the risk of CVD is rather weak and mainly caused by excess visceral fat in patients with obesity (36). Xu et al. (37) dissociated and analyzed the contribution of diabetes to the incidence of post-stent MACE by excluding those studies with diabetes patients from the MS group, and concluded that there was no significant difference in the incidence of MACE in the MS group with or without diabetes patients, which is consistent with our findings. The discrepancies between existing studies and our study may be mainly due to the dissimilarities of patient populations. Patients should be classified as at high risk even if there are no obvious blood glucose abnormalities. Therefore, MS phenotypes are important in CV risk assessment and in educating patients and physicians about preventive measures.

We found that HDL-C was associated with a neutral risk of 1-year MACE in patients with NSTEMI, as confirmed by a previous study (38). This may be due to HDL function being impaired with CVD (39). Under certain conditions, HDL would lose its protective functions (antioxidant, anti-inflammation, anti-apoptotic, and ameliorate endothelial dysfunction) and gain dysfunction, which might contribute to the inflammatory process of CVD in patients with atherosclerosis. With respect to hypertriglyceridemia, a previous study also showed that hypertriglyceridemia did not have any association to high-risk all-cause mortality in men and for CVD mortality in women (40). Hypertriglyceridemia and mortality in older adults could be due to selective survival, since individuals with higher cardiovascular risk associated with elevated triglycerides could have died earlier, while more resilient adults reached older age (41). The neutral effect of hypertension on patients with NSTEMI undergoing PCI has been previously demonstrated. Cecchi et al. conducted a study (42) on the impact of hypertension on MI patients undergoing PCI, and came to the conclusion that hypertension was not associated with either in-hospital and long-term mortality in patients with NSTEMI, which was consistent with our results. The reason for the scarce impact of hypertension on NSTEMI outcome can be ascribed to our study population, which is only the patients undergoing PCI, playing a critical role in restoring coronary artery perfusion, alleviating myocardial ischemia, and, ultimately, improving their long-term survival.

Although the subgroup analyses conducted in this study were exploratory, the results raised some important points, as limited data are available on the prognostic value of MS in different subgroups of patients with NSTEMI. Our study showed that MS was correlated with higher MACE, especially in older patients and those with LVEF of ≤40%, GRACE of >140, and MVD or hsTNT of >0.1 ng/ml. Previous studies have proven that age is a powerful predictor of adverse events after ACS (43). GRACE risk score is recommended by international guidelines to assess the risk of future ischemic events in patients with NSTEACS to facilitate evidence-based treatment in the future (44, 45). Kim et al. assumed that MVD involvement might negatively influence cardiovascular outcomes in patients with MS (46). Al Suwaidi et al. (47) found recurrent ischemia was more common in MS group, which leads to lower LVEF in patients with MS, and statistically significant difference in mortality compared with those without MS. All these conditions indicate myocardial ischemia. In practice, advanced vascular damage is often associated with the presence of MS in patients with CVD, and it is significantly associated with lipid-rich plaques in coronary arteries, which increases the risk of rupture and leads to poor outcomes (48, 49). In other words, we concluded that the impact of MS on prognosis value was evident when the amount of myocardial necrosis is greater and cardiac function is worse.

Azarfarin et al. (50) found that increased WC was associated with greater myocardial necrosis and worsening LVEF in patients with AMI. This suggested that obesity was associated with adverse cardiovascular outcomes, which also supported our finding.

## Limitations

There are several limitations in the present study. First, abdominal obesity has a better predictive value for the risk of CVD than BMI. In this paper, BMI was used to replace abdominal circumference in the diagnosis of MS, which may have a certain impact on the results. Second, the patients were followed up for only 1 years, which may have some effect on the final clinical outcome analysis. Third, considering that the patients in our study were all from China, which might limit the generalization of the fingings to other races. Last, dynamic changes in MS status may confuse the association between MS individual components and MACE.

## Conclusion

As far as we know, no previous studies have investigated the prognostic value of MS in patients with NSTEMI undergoing PCI. Our study demonstrated that MS had a negative impact on MACE in those patients. In MS component analysis, a BMI of ≥28 kg/m^2^ independently increased the occurrence of MACE. Therefore, we suggest that patients with NSTEMI should be aware of the presence of MS, especially in older patients who have higher ischemic risk. Active prevention and treatment of MS may improve its clinical efficacy by controlling abdominal obesity.

## Data Availability Statement

The original contributions presented in the study are included in the article/supplementary material, further inquiries can be directed to the corresponding author/s.

## Ethics Statement

The study was approved by the Ethics Committee (No. 2018KY-010-01). The patients/participants provided their written informed consent to participate in this study.

## Author Contributions

L-HZ, YL, and JG: conceptualization and design. J-YX, J-XW, and X-WL: resources. L-HZ, YL, and ZC: formal analysis and data curation. L-HZ: writing—original draft preparation. JG: project administration and funding acquisition. All authors have read and agreed to the published version of the manuscript.

## Funding

This work was supported, in part, by the Key Project of Scientific and Technological Support Plan of Tianjin, China (No. 20YFZCSY00820).

## Conflict of Interest

The authors declare that the research was conducted in the absence of any commercial or financial relationships that could be construed as a potential conflict of interest.

## Publisher's Note

All claims expressed in this article are solely those of the authors and do not necessarily represent those of their affiliated organizations, or those of the publisher, the editors and the reviewers. Any product that may be evaluated in this article, or claim that may be made by its manufacturer, is not guaranteed or endorsed by the publisher.
